# Self-Care Strategies and Job Satisfaction in Pediatricians: What We Can Do to Prevent Burnout—Results of a Nationwide Survey

**DOI:** 10.3389/fped.2021.722356

**Published:** 2021-08-31

**Authors:** Ulrike Leiss, Andrea Schiller, Jonathan Fries, Peter Voitl, Andreas Peyrl

**Affiliations:** ^1^Department of Pediatrics, Medical University of Vienna, Vienna, Austria; ^2^Comprehensive Center for Pediatrics, Medical University of Vienna, Vienna, Austria; ^3^Department of Pediatrics and Neonatology, St. Josef Hospital Vienna, Vienna, Austria; ^4^Department of Developmental and Educational Psychology, Faculty of Psychology, University of Vienna, Vienna, Austria; ^5^First Vienna Medical Care Center, Vienna, Austria

**Keywords:** well-being, job satisfaction, burnout, self-care, individual interventions, organizational interventions

## Abstract

**Introduction:** Working in the clinical field can be a demanding experience. While reports indicate escalating burnout rates among physicians, further investigation about what physicians can do to prevent burnout is necessary. Our objective was to assess self-care levels among pediatricians and the correlation with job satisfaction in order to subsequently identify protective factors.

**Methods:** In this nationwide, cross-sectional study, a web-based survey was distributed to all Austrian pediatricians via a mailing list of the Austrian Society of Pediatrics. Self-care was measured with a modified Professional Self-Care Scale (PSCS), which consisted of nine items on a four-point Likert scale (from 1, “totally disagree,” to 4, “totally agree”). Additional items addressed job satisfaction, peer support, and access to professional coaching.

**Results:** The survey was distributed to 1,450 mailing list contacts, a total of 309 Austrian pediatricians completed the survey (21%). “Family” (*M* = 3.3) and “Friends” (*M* = 3.1) were the most highly rated self-care strategies. We found significant differences between pediatricians under 35 years and those aged 50 years and above (*z* = −4.21, *p* < 0.001). Peer support appeared to impact job satisfaction substantially. We found differences between pediatricians who frequently talk to colleagues about difficult situations, those who sometimes do so, and those who never do (*p* < 0.001), with a linear trend indicating increased job satisfaction with more frequent peer support (*p* < 0.001).

**Conclusions:** Among all self-care strategies, a stable network of family and friends was highest rated, followed by balanced nutrition. Younger, male pediatricians working in hospital showed to be a vulnerable group with regard to overall self-care. Moreover, higher self-care values were found among those pediatricians who reported to receive peer support on a regular basis. We suggest combined organizational and individual interventions to promote pediatricians' well-being. Organizations should provide the possibility to select a well-balanced diet as well as space and time to consume food and cultivate a work environment that enables communication among peers and facilitates professional coaching. On the personal level, we want to encourage pediatricians to talk to trusted colleagues in challenging clinical situations and to consider working with a professional coach.

## Introduction

Working in the clinical field as a physician can imply long working hours with high stress levels, frequent exposure to death, sleep deprivation, and lack of leisure time. Today, the medical profession demands compassionate presence and collaborative decision-making with patients, above all through active listening and respect of their choices, priorities, beliefs, and values. All of these circumstances create an area of conflict between reality and expectations.

Hence, the patient is not the only person in the consulting room. The health risks of working in a demanding area are well-documented (1–4). One index of these problems—burnout—has received a great deal of attention in recent years.

Physician burnout is a distinct work-related syndrome, resulting from tensions that arise between the physician and his or her work environment (5, 6). The concept of burnout syndrome entails emotional exhaustion, cynicism, and a sense of inefficacy. Consequences of burnout are negative effects on patient care, increased patient dissatisfaction, but also increased medical errors, and personal and professional issues (6).

Burnout has reached epidemic levels among physicians, regardless of their specialty (7, 8). In a recent survey among pediatric residents, 39% of respondents experienced burnout, associated with self-reported negative patient care attitudes and behaviors (9). Moreover, burnout represents a financial burden for institutions (10, 11).

Despite the recent public interest in this subject and the literature substantiating the impact burnout has on the individual's health, few studies discussed strategies to prevent burnout in the pediatric field. This study specifically focused on self-care strategies and job satisfaction rather than burnout, based on a resource- and action-oriented concept. It has been documented that both individual-focused and organizational interventions can mitigate physician burnout (12). Individual-focused strategies consisting of self-care training can result in clinically meaningful reductions in burnout among physicians and allow them to succeed in their demanding job (12, 13).

Self-care is a multi-faceted construct. It encompasses physical self-care, which includes activities that help maintain a healthy body—inner self-care, related to practices for a healthy mind, and social self-care, involving activities that maintain social health (14). It should be emphasized that adequate self-care can be an essential part of the therapeutic activity, enabling physicians to care for their patients in a sustainable way with greater compassion, sensitivity, effectiveness, and empathy (15, 16).

Therefore, this study aimed to assess self-care levels in Austrian pediatricians in a nationwide survey and the correlation with job satisfaction to provide concrete recommendations for an improved well-being.

## Methods

### Participants and Survey Instrument

Austrian pediatricians were recruited to participate in this nationwide cross-sectional study through an email with a link to a Google Forms electronic survey. Participation was elective, and responses were anonymous. The survey was distributed to 1,450 mailing list contacts of the Austrian Society of Pediatrics, a reminder email was sent after 6 weeks.

In order to specifically measure self-care strategies instead of burnout we used a modified Professional Self-Care Scale (PSCS), which assesses three dimensions of professionals' self-care strategies: physical self-care, inner self-care, and social self-care (14). The three dimensions contribute to the overall self-care factor. The modified survey instrument consisted of nine items on a four-point Likert scale (from 1, “totally disagree,” to 4, “totally agree”) to estimate the extent to which pediatricians implement self-care strategies (see Supplementary Material). Additionally, the electronic survey collected socio-demographic data and investigated job satisfaction, peer support, and current or former access to professional coaching in a clinical context, individually or in the team, in order to reflect on one's own behavior and actions as well as ways of reacting when working with patients, but also on team processes. The study on anonymous pediatricians was considered exempt from the requirement to undergo review by the Ethics Committee of the Medical University of Vienna.

### Data Analysis

We used descriptive statistics to describe our sample. Since dependent variables were ordinal-scale survey responses, or mean scores derived from these, we used non-parametric statistics to test our hypotheses. For two-group comparisons, the Wilcoxon rank-sum test was applied; for comparisons of three or more groups, the Kruskal-Wallis test was carried out. To further parse the results of the multiple-group comparisons, we used Dunn's test with Bonferroni correction. To assess linear trends, we applied two-sided non-parametric Jonckheere-Terpstra tests for ordinal data with 1,000 permutations of the *p*-value. We chose to report effect sizes whenever possible. Due to the ordinal nature of the data, we used non-parametric effect sizes, e.g., epsilon squared for Kruskal-Wallis tests.

We hypothesized that a person's job satisfaction could be predicted by the degree to which they applied self-care strategies. To this end, we carried out a multiple ordinal regression analysis.

All analyses were performed using the statistical programming environment R 3.6; graphics were created using the R package ggplot 2.

## Results

A total of 309 pediatricians completed the online survey (Table 1). Considering the distribution of the survey by the society's mailing list we achieved a fairly high response rate of 21% compared to studies that used a similar survey distribution mode (17–19). The majority of pediatricians that completed the survey were general pediatricians (45%). Of the participants completing the survey, 65% worked in the hospital, 25% in private practice, and 10% both in hospital and private practice. This is similar to the overall population of Austrian pediatricians, where 49% work in the hospital, 38% in private practice, and 13% both in hospital and private practice.

**Table 1:** Pediatricians' demographics (*N* = 309).

**Demographic factors**					**Missing**
*Sex, N = 309*	**Female**	**Male**			
*n* (%)	175 (57%)	134 (43%)			
*Age, N = 304*	** <35 years**	**35–50 years**	**>50 years**		
*n* (%)	69 (22%)	125 (40%)	110 (36%)		5 (2%)
*Setting, N = 304 n* (%)^a^	**Hospital**	**Private practice**	**Hospital and private practice**	**Other**	
	198 (64%)	67 (22%)	29 (9%)	10 (3%)	5 (2%)
*Providing care to life-limiting ill/dying children, N = 306 n* (%)	**Yes**	**No**			
	243 (79%)	63 (20%)			2 (1%)

a *The proportion of pediatricians in general pediatrics was highest (39%), followed by neonatology and intensive care (14%). In total, survey respondents came from 15 subspecialties*.

### Self-Care Strategies

**Figure 1:**
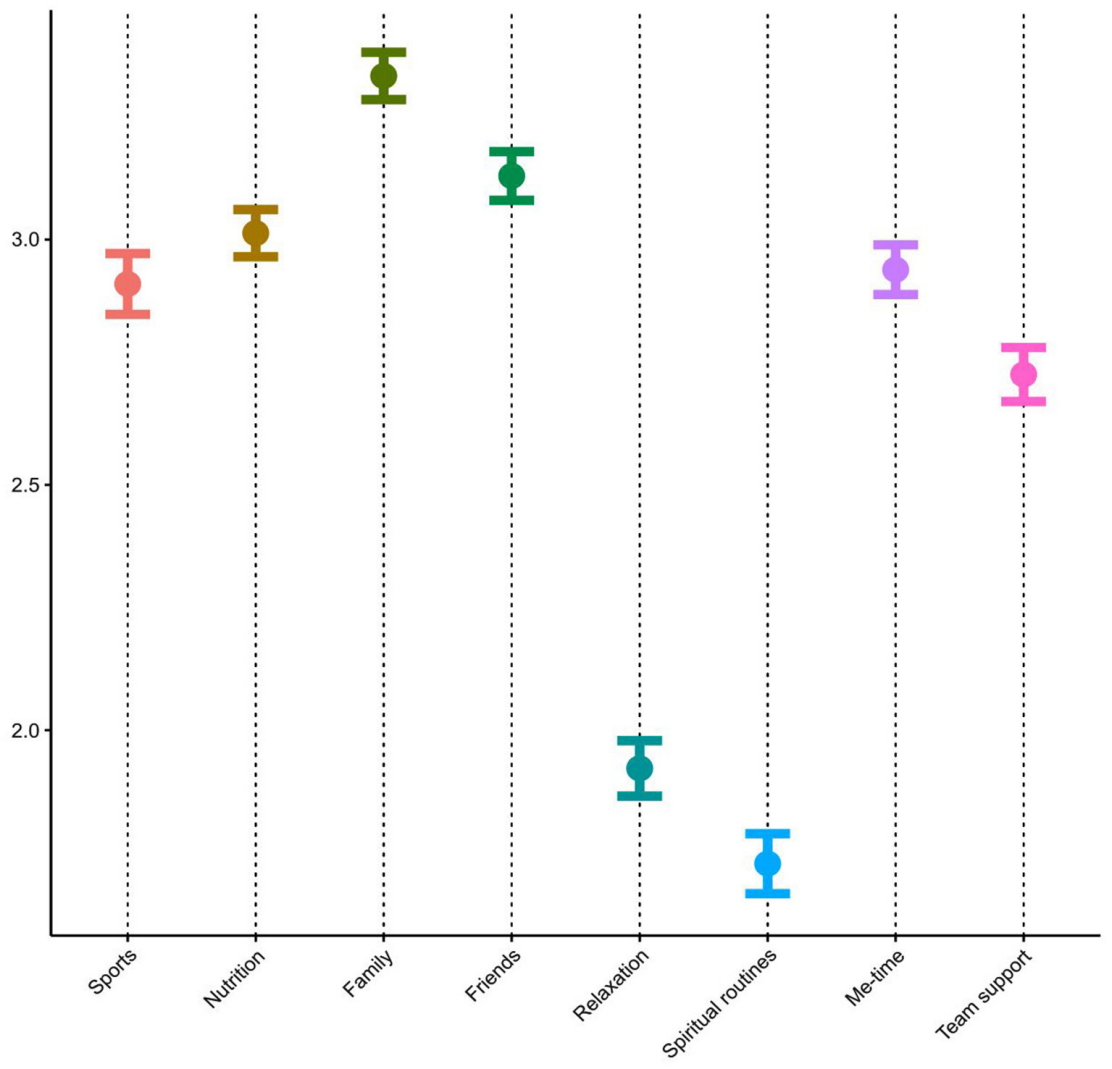
Use of self-care strategies (self-report on a four-point Likert scale from 1, “totally disagree,” to 4, “totally agree”).

On average, “Family” (*M* = 3.3) and “Friends” (*M* = 3.1) were the most highly rated self-care strategies, while “Relaxation” (*M* = 1.9) and “Spiritual Routines” (*M* = 1.7) received the lowest ratings on a four-point Likert scale (Figure 1).

To generate a general estimate of self-care, we calculated a mean score across ratings in all eight self-care strategies for each participant. We found a moderate gender-based difference with regard to the self-care score (*p* = 0.02), with women achieving higher overall scores.

**Figure 2:**
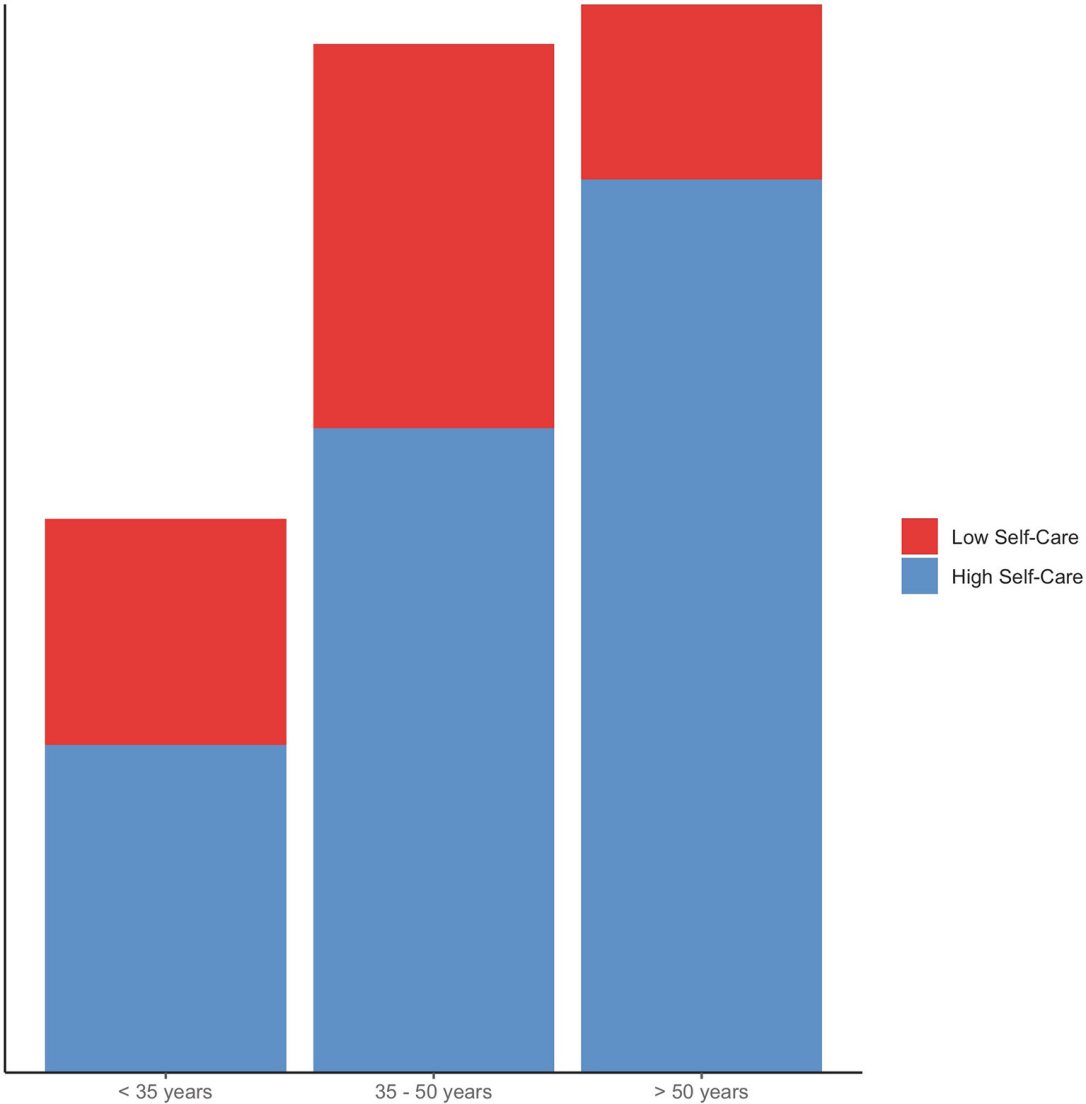
Self-care by age group. High and low self-care categories were formed using a mean split.

We also found significant differences between pediatricians under 35 years and those aged 50 years and above (*z* = −4.21, *p* < 0.001) as well as between the 35–50 and >50 age groups (Dunn's test *z* = 3.33, *p* < 0.001) and could therefore identify a significant linear trend, with the use of self-care strategies progressively increasing over age (*JT* = 18,367, *p* < 0.001) (Figure 2). The degrees of self-care varied depending on the pediatricians' workplace: physicians who work in private practices reported using self-care strategies significantly more often than those employed in hospitals (Dunn's test *z* = −3.21, *p* < 0.001). Also, 57 participants (17%) cited additional personal self-care strategies, such as: making music, enjoying nature, gardening, traveling, cultural activities, cooking, wellness, and psychotherapy. However, this group did not differ from the other respondents with regard to the self-care mean score (*W* = 6,877, *p* = 0.33, *r* = −0.06).

### Professional Coaching and Self-Care

**Figure 3:**
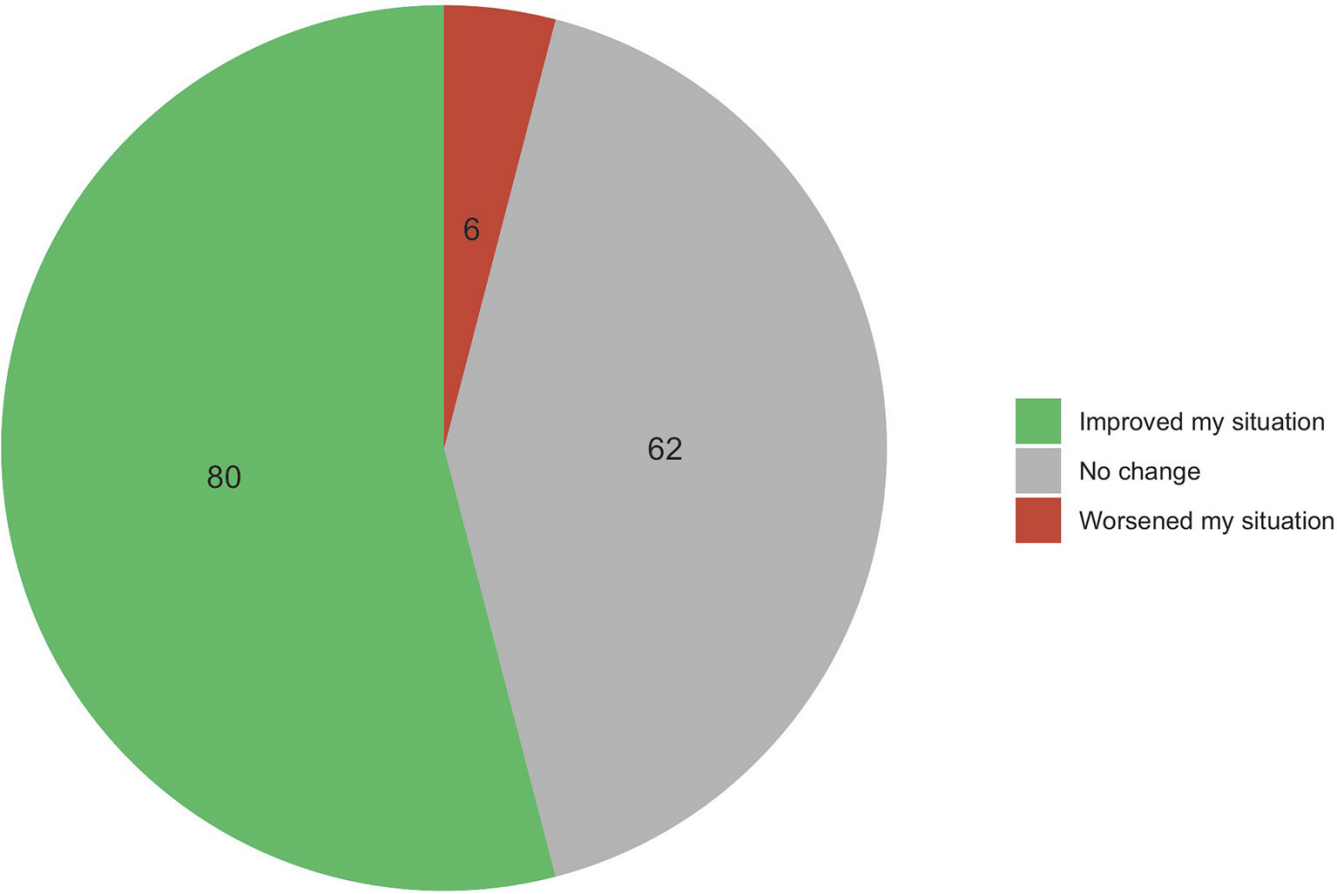
Impact of professional coaching on pediatricians' job satisfaction.

More than half of the respondents (52%) reported that they had never participated in professional coaching before. Nevertheless, 81% declared being willing to do so if offered to them. On the other hand, 148 pediatricians (48%) had previously attended professional coaching. More than half of them (54%) reported improvements, 41% experienced no changes, 4% stated that their situation worsened due to professional coaching (see Figure 3). Remarkably, only 22% were participating in a professional coaching at the time of the survey (with funding by the institution for 51% of them or 11% of respondents).

### Peer-Support and Self-Care

Almost two-thirds of survey-respondents (196/63%) declared consulting colleagues in challenging situations regularly, 32% reported doing so occasionally, 4% never consulted colleagues. We found significant differences in their use of self-care strategies (Kruskal-Wallis χ^2^ = 13.91, *df* = 2, < 0.001, epsilon-squared = 0.05), and the results revealed a linear trend with the lowest self-care values for pediatricians who never receive peer support and the highest self-care values for those who reported to receive peer support on a regular basis (Jonckheere-Terpstra test *JT* = 14,552, *p* < 0.001).

### Job Satisfaction

**Figure 4:**
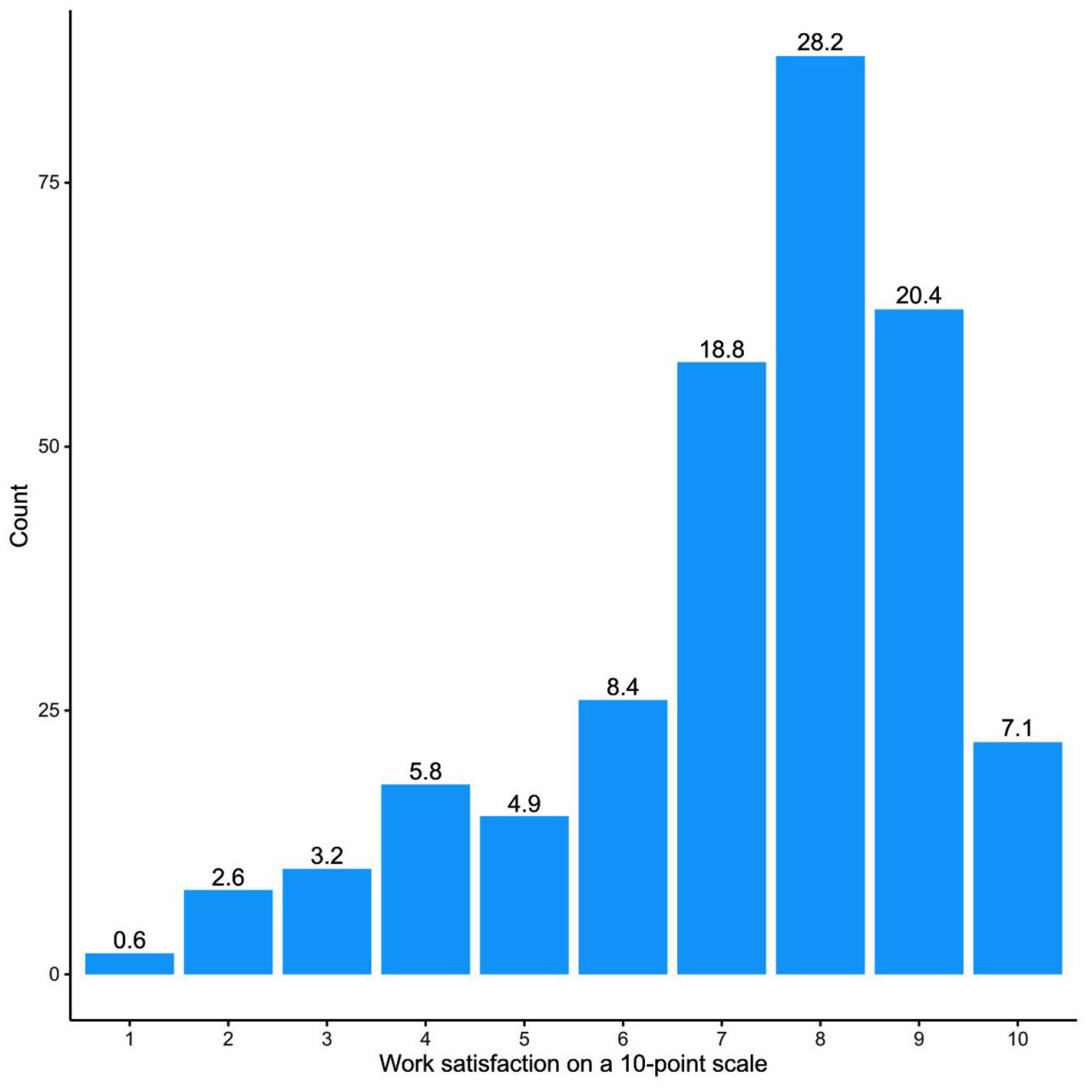
Job satisfaction of pediatricians in the survey (*N* = 309).

With regard to their job satisfaction, 75% of the participating pediatricians gave a 7 or higher rating on a 10-point scale (10 meaning the highest satisfaction, Figure 4).

We found significant job satisfaction differences between age groups (Kruskal-Wallis χ^2^ = 10.78, *df* = 2, *p* < 0.001, epsilon-squared = 0.04). Pediatricians younger than 35 manifested significantly lower job satisfaction than the age group above 50 years (Dunn's test *z* = −3.28, *p* < 0.001), revealing a significant linear trend that estimates job satisfaction to increase with age progressively (*JT* = 17,560, *p* < 0.001). Moreover, we found significant differences in job satisfaction between work environments (Kruskal-Wallis χ^2^ = 14.10, *df* = 3, *p* < 0.001, epsilon-squared = 0.05). Pediatricians who work in hospitals were significantly less satisfied than colleagues who work in private practices (Dunn's test *z* = −3.64, *p* < 0.001). We could not identify a statistical correlation between the former or current participation in professional coaching and job satisfaction. Job satisfaction did not seem to differ between doctors who provide care to children with life-limiting diseases and those who do not (*W* = 7146.5, *p* = 0.40, *r* = −0.05).

**Figure 5:**
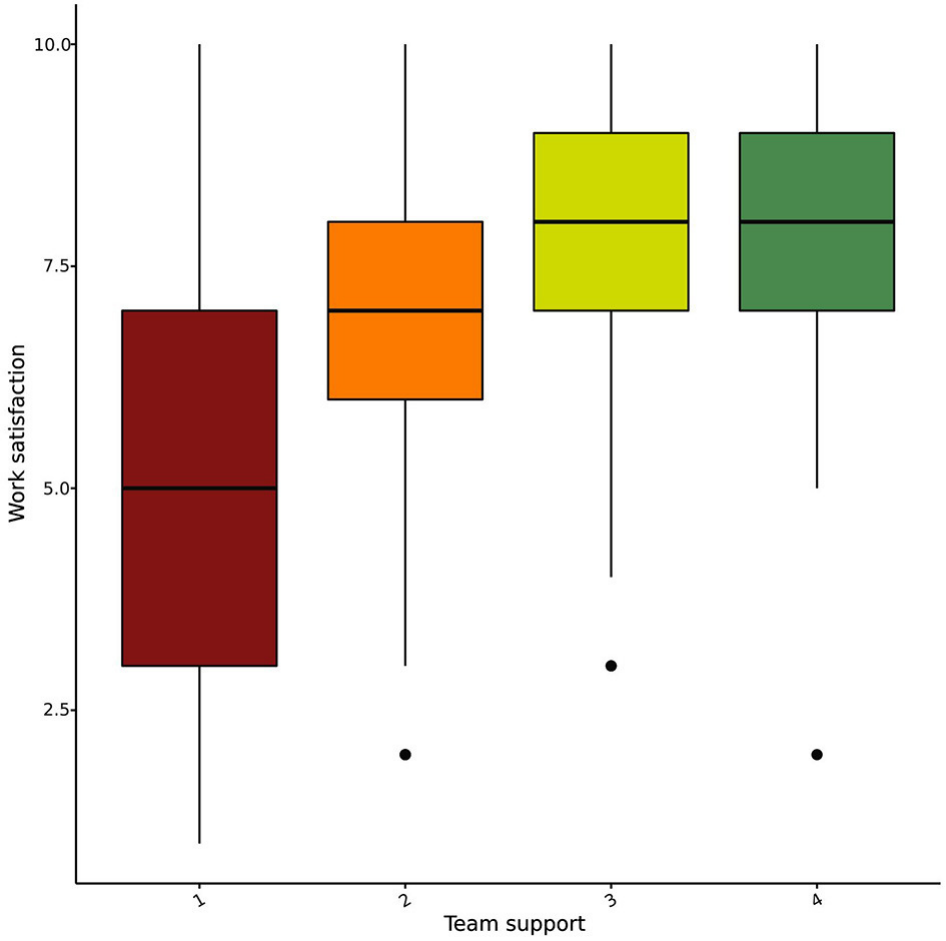
Job satisfaction grouped by the level of team support experienced by pediatricians.

Peer support appeared to impact job satisfaction substantially. We found differences between pediatricians who frequently talk to colleagues about difficult situations, those who sometimes do so, and those who never do (*p* < 0.001), with a linear trend indicating increased job satisfaction with more frequent peer support (*p* < 0.001) (Figure 5).

### Association Between Job Satisfaction and Self-Care

**Figure 6:**
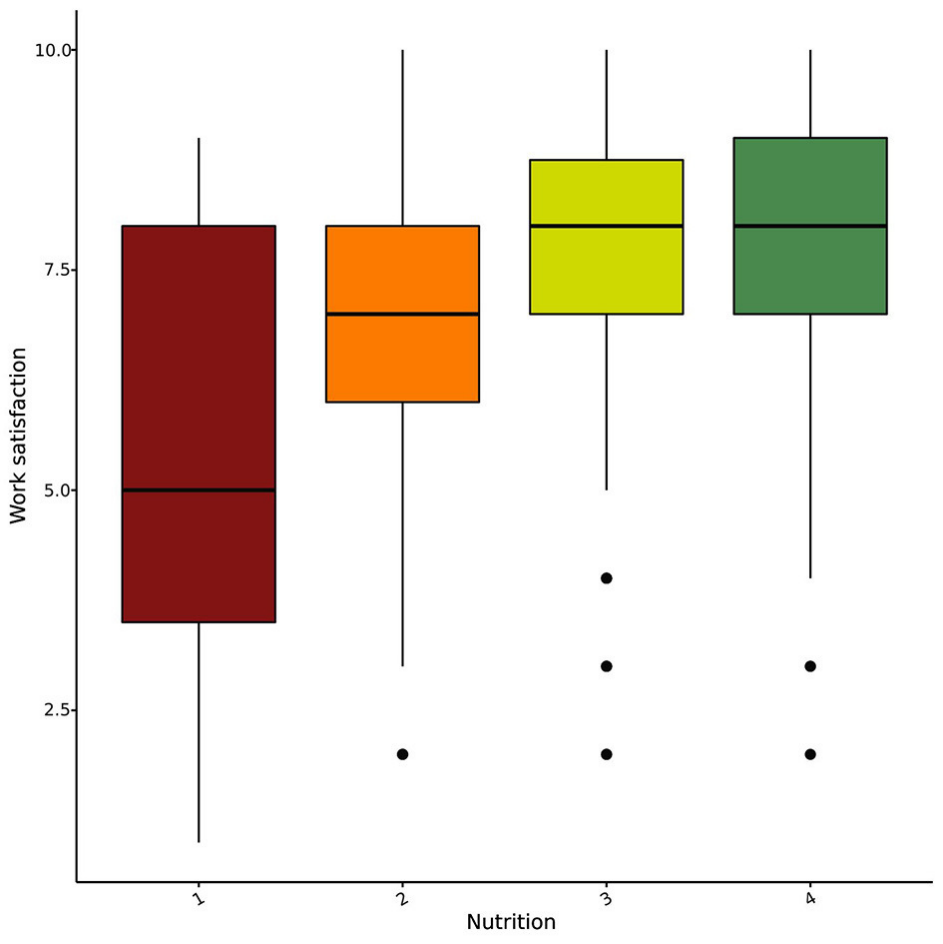
Job satisfaction grouped by the self-rated quality of respondents' nutrition.

**Figure 7:**
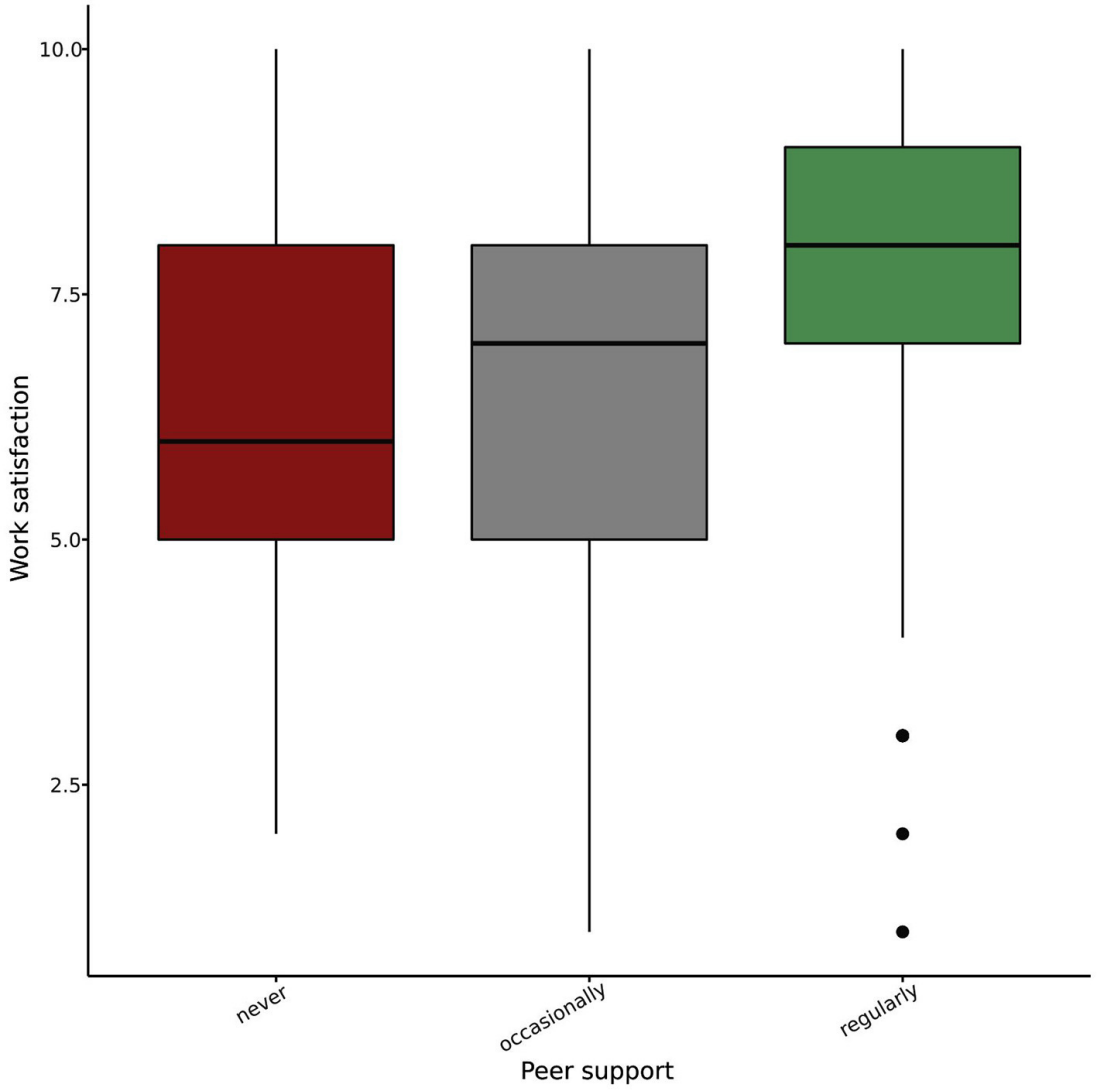
Job satisfaction grouped by the frequency by which pediatricians report to receive peer support in demanding clinical situations.

Job satisfaction and the self-care mean score were significantly correlated at a medium effect size (Spearman *rho* = 0.42, *p* < 0.001). We hypothesized that variation in job satisfaction could be accounted for the variation in the use of self-care strategies. To test this hypothesis, we ran a multiple ordinal logistic regression model. All eight self-care strategies were included as individual predictors while job satisfaction was defined as the criterion variable. The factors sex, age, and work environment are categorical variables in our dataset, therefore we did not include these factors in the regression framework. The model explained 32% of the variance in job satisfaction (Nagelkerke pseudo *r*-squared), which indicates a fairly good fit (20). Nutrition, friendship, and peer support were the most influential variables in the model (Table 2; Figures 6, 7).

**Table 2:** Use of self-care strategies and job satisfaction—results of logistic ordinal regression model.

**Predictor**	**Logistic coefficient**	***SE***	***p*-value**	**Odds ratio**
Sports	−0.02	0.11	0.84	0.98
Nutrition	0.53	0.16	0.00^**^	1.7
Family	0.21	0.14	0.14	1.23
Friends	0.44	0.14	0.00^**^	1.56
Relaxation	0.14	0.12	0.26	1.15
Spiritual routines	−0.11	0.11	0.32	0.89
Me-time	0.12	0.13	0.32	1.13
Team support	0.77	0.13	0.00^**^	2.17

** *p-value smaller than 0.00*.

## Discussion

To the best of our knowledge, this is the first study addressing well-being, self-care strategies, and self-reported job satisfaction among pediatricians through a nationwide survey. Currently, we know very little about what strategies promote physicians' well-being, which is related to the fact that this aspect is not part of physicians' professional training and is typically not listed among their top priorities.

At the same time, increasing evidence suggests that physician burnout influences the quality of care (9, 21, 22) and can contribute to medical errors (21, 22). Physician burnout is now considered a major problem in the health-care system, for its prevalence now exceeds 50%. Astonishingly, medical institutions tolerate the fact that about half of their personnel show signs or symptoms of burnout (7–9, 21, 23, 24).

Moreover, recent studies have investigated the economic burden associated with physician burnout, suggesting that the attributable expenses are, indeed, substantial (10, 11). Although organizations should support interventions to prevent and alleviate burnout for altruistic reasons, this will unlikely happen without the prospect of potential economic benefits. Initiatives may improve staff retention, reduce absenteeism, boost employee morale and job satisfaction, mitigate conflicts, and encourage physicians to be present, empathic, and efficient (4).

The present study was designed as a resource-oriented approach, focusing on self-care strategies that nurture well-being and job satisfaction.

Data analysis revealed balanced nutrition, friendship, and peer-support as protective self-care strategies that had a positive effect on job satisfaction. The use of self-care strategies progressively increases with age, and is more common among those who work in private practices and women.

Conversely, younger physicians who work in a hospital setting and do not turn to colleagues for peer-support achieved lower self-care scores and were significantly less satisfied with their work, therefore being a risk group.

Our study corroborates previous findings associating poorer job satisfaction with infrequent exercise and higher fast-food consumption, which can contribute to burnout (25). A poor diet and increased fast-food consumption have been associated with burnout, whereas a self-reported healthy diet is a protective factor (25–27). When participants in a survey among trauma surgeons reported consuming a healthy diet, they also achieved a better work-life balance (28). Our study corroborates previous findings, relating a worse work-life balance and burnout with infrequent exercise and higher fast-food consumption (25). We found that a well-balanced diet was associated with better job satisfaction. The possibility to select a well-balanced diet is a modifiable factor with regard to burnout risk (29).

Burnout rates are persistently high among residents (mostly young physicians in the hospital setting), reflecting the multifactorial nature of job-related stress among doctors-in-training (9, 24). The work of residents includes high work intensity, responsibility for patients, participation in life-and-death decision-making, witnessing tragedy, but also lack of control over schedules and the cumulative effect of long duty-hours (24). In addition, younger physicians were more prone to burnout, reported more stressors and manifestations of stress, and fewer coping mechanisms than their colleagues with more years of experience (30–32), making younger age an independent risk factor for components of burnout (30). Along with results from previous studies, our data highlight the importance of actively supporting young physicians in the hospital setting and facilitating their access to protective self-care strategies.

Our findings regarding peer support and professional coaching provide insight on the association with job satisfaction, with implications for burnout and well-being. Awareness of individual strengths and limitations is an essential resource for physicians. With respect to peer support, we found that pediatricians who frequently talk about difficult clinical situations with their colleagues better use self-care strategies and have greater job satisfaction. Previous research also confirms that sharing knowledge, experience, as well as emotional and social support could improve career satisfaction and reduce burnout (33, 34). For this purpose, some medical institutions schedule regular meetings for reflection and feedback on patients' care (3, 35, 36).

Finding a trusted colleague to discuss difficult clinical situations can be crucial to reduce isolation and build a support network (3). Participants in a facilitated small-group intervention experienced significant improvement in meaning, empowerment, and engagement (34). Additionally, multidisciplinary teams for cancer services with shared leadership of their clinical decision-making have been proven to be most effective (37). Therefore, our study's results on peer support as a protective self-care strategy with positive effects on job satisfaction are very encouraging.

Professional coaching is a viable alternative to conduct clinical observation on difficult cases (38–41). Our results regarding professional coaching were somehow ambiguous as more than half of the respondents reported that they had never participated in professional coaching before. However, although approximately 80% affirmed they would participate, if offered to them, only 22% were attending professional coaching at the time of the survey. Apart from that, we found that professional coaching has helped more than half of the recipients handle their professional life. In a previous randomized clinical trial, professional coaching participants had a significant reduction in emotional exhaustion and burnout symptoms, as well as improvements in overall quality of life and resilience (31). Even brief interventions could have a meaningful impact, as demonstrated in a 5-h mindfulness-based self-care curriculum for an interprofessional group of palliative care providers. The reported changes were sustained even 7 months after completion of the series (42).

However, as doctors are typically reluctant to seek help for themselves, offering low-key access to professional coaching remains an urgent matter (43).

Our study has several important strengths. To our knowledge, this is the first nationwide survey exploring the relationship between self-care strategies, well-being, and job satisfaction among pediatricians. The observed association was statistically significant and large enough to suggest that the correlation of self-care strategies, peer support, professional coaching, and job satisfaction could be meaningful. Lastly, our sample, including specialists working in hospitals and private practices, is relatively heterogeneous. Therefore, these results are likely to be relevant to other medical specialties and health care providers.

There are some limitations to our study. Although the response rate obtained in this study is comparable to that of other medical surveys, response bias remains a possibility. Age was used as a surrogate marker as time in practice. Moreover, our results rely on self-report instead of behavioral data.

## Conclusion

Our nationwide survey among pediatricians reveals that well-balanced nutrition, friendship maintenance, and peer-support are protective self-care strategies that have a positive effect on job satisfaction. We noticed that younger pediatricians working in hospitals seem to be a vulnerable group.

Therefore, we propose a combined approach of organizational and personal interventions, in which physicians and health care organizations share the responsibility to promote physician well-being. Organizations should incorporate healthy meal options and especially space and time to consume food and support the working atmosphere of employees to enable conversations among peers (i.e., support joint social activities). Ultimately, organizations should enable low-key access to professional coaching. We want to encourage physicians to resort to individual interventions such as talking to trusted colleagues on a regular basis and working with a professional coach to improve job satisfaction and personal well-being.

## Data Availability Statement

The original contributions presented in the study are included in the article/Supplementary Material, further inquiries can be directed to the corresponding author/s.

## Ethics Statement

Ethical review and approval was not required for the study on human participants in accordance with the local legislation and institutional requirements. Written informed consent for participation was not required for this study in accordance with the national legislation and the institutional requirements.

## Author Contributions

UL, AS, and AP: study design and design of data collection instruments. UL, AS, AP, and PV: data collection. UL and JF: data analysis. UL and AP: writing of the manuscript. UL, AS, PV, JF, and AP: editing and revision of the manuscript. All authors approved the final manuscript as submitted and agree to be accountable for all aspects of the work.

## Conflict of Interest

The authors declare that the research was conducted in the absence of any commercial or financial relationships that could be construed as a potential conflict of interest.

## Publisher's Note

All claims expressed in this article are solely those of the authors and do not necessarily represent those of their affiliated organizations, or those of the publisher, the editors and the reviewers. Any product that may be evaluated in this article, or claim that may be made by its manufacturer, is not guaranteed or endorsed by the publisher.
