# Supplementary material for: Self-Care Strategies and Job Satisfaction in Pediatricians: What We Can Do to Prevent Burnout—Results of a Nationwide Survey
Source: Front Pediatr. 2021 Aug 31;9:722356. doi: 10.3389/fped.2021.722356 (PMC8438418; doi:10.3389/fped.2021.722356)
Supplement: Supplementary file 1 [file Data_Sheet_1.DOCX]

**Supplemental Data**

**Survey Instrument**

We used a modified Professional Self-Care Scale (PSCS), which assesses three dimensions of professionals’ self-care strategies: physical self-care, inner self-care, and social self-care (questions 1-9).^14^ The modified survey instrument consisted of nine items on a 4-point Likert scale (from 1, “totally disagree,” to 4, “totally agree”) to estimate the extent to which pediatricians implement self-care strategies. Additionally, the electronic survey collected socio-demographic data and investigated job satisfaction, peer support, and current or former access to professional coaching.

*Question 1:* I do exercise on a regular basis.

*Question 2:* I usually follow a balanced diet.

*Question 3:* I believe that my family relations outside work are satisfactory.

*Question 4:* I believe that my friendships outside work are satisfactory.

*Question 5:* I practice activities that help me to relax.

*Question 6:* I get actively involved in spiritual practice (for example, meditation, oration, yoga, …).

*Question 7:* I am constant.

*Question 8:* When I feel emotionally overloaded I try to find time for my own care.

*Question 9:* When I feel overwhelmed by a clinical situation I feel that I can support on my team in order to elaborate this experience.

*Question 10:* Have you ever participated in professional coaching?

*Question 10a:* Would you participate in professional coaching if offered?

*Question 10b:* Do you currently participate in professional coaching?

*Question 10c:* Funding of professional coaching: Institution/private/both

*Question 10d:* What were your experiences with professional coaching?

Did improve my situation.

My situation did not change.

My situation worsened.

*Question 11:* Do you talk with colleagues about difficult clinical situations?

Yes/No/Sometimes

*Question 12:* When you go to work in the morning, how would you rate your job satisfaction on a 10 point scale (1 not satisfied, 10 very satisfied)?

*Question 13:* Gender

*Question 14:* Age

*Question 15:* Working environment: Hospital/Private Practice/Both

*Question 16:* What is your main subspecialty?

General pediatrics

Pediatric endocrinology

Developmental pediatrics

Pediatric gastroenterology

Pediatric infectious diseases

Adolescent medicine

Neonatology and pediatric critical care

Pediatric nephrology

Neuropediatrics

Pediatric hematology-oncology

Pediatric cardiology

Pediatric pulmonary medicine/allergy

Pediatric psychsomatic medicine

Pediatric sonography

Pediatric metabolism disorders

*Question 17:* Do you provide care to children with life-limiting diseases or dying chilren?

Yes, >10/year; Yes, 10/year; Yes, sometimes; No

*Question 18:* Do you apply self-care strategies that were not mentioned above? Which strategies? (optional)
